# Supplementary material for: A novel home-use culture mechanism for identifying microbial load in urine samples
Source: PLoS One. 2023 May 31;18(5):e0285881. doi: 10.1371/journal.pone.0285881 (PMC10231770; doi:10.1371/journal.pone.0285881)

**Supplementary Fig 1. Growth of E.coli on HiChrome UTI agar delivered as per experimental design**

Growth of cultured E.coli on HiChrome UTI agar applied using various methods i.e. top streak, bottom streak and bottom drop and over serial dilutions (a). Comparison of CFU/ml obtained from different application methods and using different quantification methods. CFU/ml was significantly different (p<0.05) between all dilutions for both the quantification methods as well as for all methods of application. CFU/ml calculated manually and by OpenCFU were not significantly different (p>0.1).


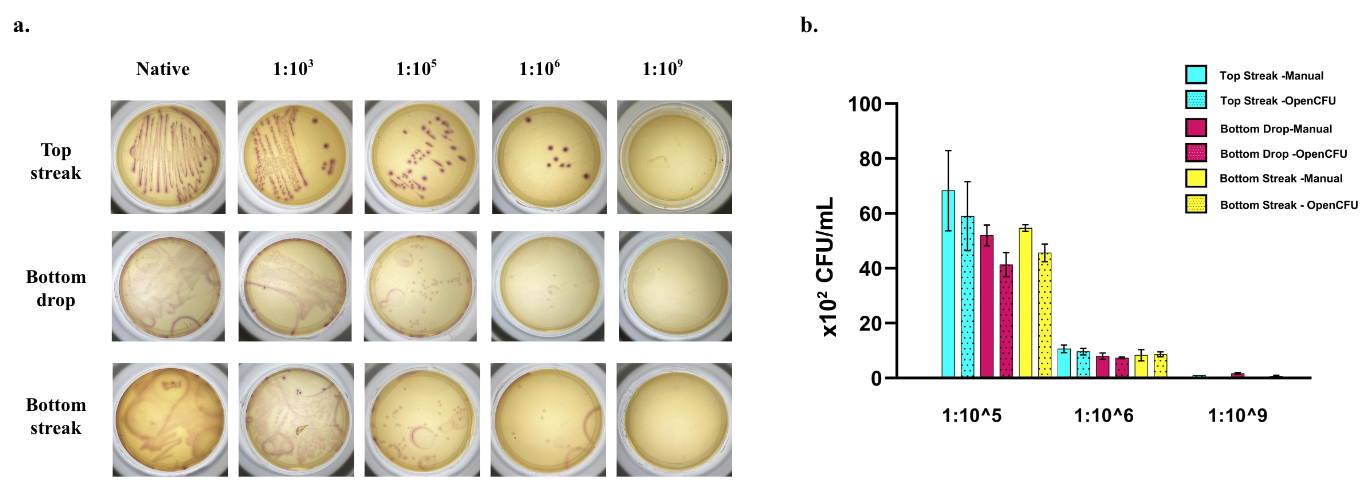

Supplement: S1 Fig — (DOCX) [file pone.0285881.s001.docx]
