# Supplementary material for: A novel home-use culture mechanism for identifying microbial load in urine samples
Source: PLoS One. 2023 May 31;18(5):e0285881. doi: 10.1371/journal.pone.0285881 (PMC10231770; doi:10.1371/journal.pone.0285881)

**Supplementary Fig 2. Membrane based culture delivery and corresponding growth of E.coli at different temperatures in HiChrome UTI agar**

Growth of cultured E.coli on HiChrome agar at 37℃ for 16 hours and at 25℃ for 30 hours with the sample delivered using the membrane (a). Visual comparison of CFU obtained manually for both temperature conditions (b). CFU was significantly different (p<0.05) between all dilutions in which colonies could be counted except between 1:10^5^ and 1:10^6^ at 25℃ (p=0.22). CFU calculated for both conditions were not significantly different in any dilution (p>0.1).


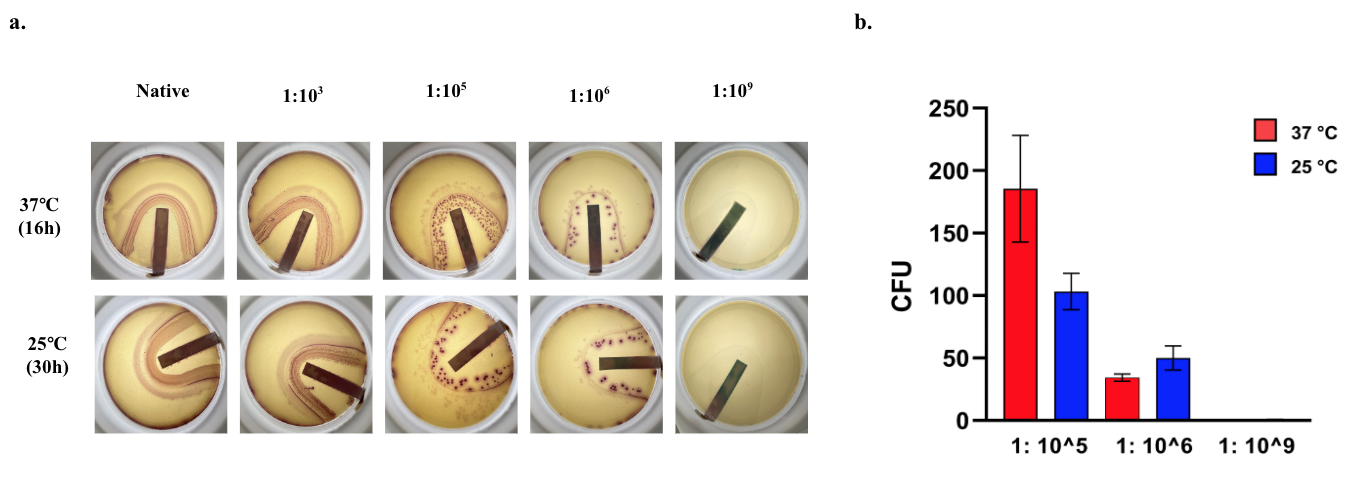

Supplement: S2 Fig — (DOCX) [file pone.0285881.s002.docx]
