# Supplementary material for: A novel home-use culture mechanism for identifying microbial load in urine samples
Source: PLoS One. 2023 May 31;18(5):e0285881. doi: 10.1371/journal.pone.0285881 (PMC10231770; doi:10.1371/journal.pone.0285881)
Supplement: Attachment — Submitted filename: Response_to_reviewers.docx [file pone.0285881.s003.docx]

Dear Professor Gadekallu,

Thank you so much for giving us an opportunity to make revision to our manuscript. We are really grateful to all the reviewers and editors for their comments and support. We have now addressed the major comments raised by the reviewers and have edited our manuscript accordingly. The changes have been tracked in the revision that we are submitting. In addition, we are also submitting a clean copy of the manuscript with the changes made. We hope that the changes are satisfactory to the editors. Below is a detailed description of each comment and our response to that:

Reviewer #1: In this work, the author proposed a novel home-use culture mechanism for identifying microbial load in urine samples. The comments are listed as follows:

1. The contribution should be summarized in several sentences in brief, which can help the reader better understand the significance of this paper. - We are grateful to the reviewer for this comment. We have changed the last paragraph of our introduction in order to summarize the findings of this paper. We believe that this fits well with the flow of the introduction.

2. The existing works should be summarized and presented with the time or logic order rather than the direct illustration, the reviewer thinks that one table for the current efforts comparison may be better.

3. What are the shortcomings or research gaps for the current related work? I think the authors should highlight them at the end of each subsection of the related work section. - We would like to thank the reviewer for this comment. We have added the shortcomings of the existing works and have highlighted them in the introduction for reference. In addition, we have also added a detailed description of the research gaps of our work in the discussion section.

4. Where is the source of the adopted data? Please add some explanations. - We are grateful to the reviewer for pointing this out. We have added the source of the data used in the materials and methods and have also explained the parameters.

5. What are the detailed parameter settings for the adopted neural networks? The authors should add more details to the manuscript. - Again, we are thankful for this comment. We agree that the parameter settings were not clearly explained and we have added the setting details in the materials and methods.

6. To broaden the scope of this paper, the authors should refer to some papers. For example, AoI optimization in the UAV-aided traffic monitoring network under attack: A Stackelberg game viewpoint; BSIF: Blockchain-based Secure, Interactive, and Fair Mobile Crowdsensing;

Mixed Game-based AoI Optimization for Combating COVID-19 with AI Bots; Data Freshness Optimization Under CAA in the UAV-Aided MECN: A Potential Game Perspective. - We would like to thank the reviewer for this comment. While we have used the papers to structure the manuscript now, we feel that the papers cannot be referred to since the scope of these works are very different from our current research.

7. This paper needs to be carefully proofed and polished before being accepted.

Reviewer #2: The work is interesting and scientifically good. However, the structure of the article should be redesigned

1)The abstract should focus on the problem and how it is solved. - We would like to thank the reviewer for this comment. We agree that the abstract in our initial submission was limited. However, we have made it more elaborate to highlight the problem and how our proposed method alleviates the issue.

2)The introduction part requires related and motivation. - We are thankful to the reviewer for suggesting this. We have edited the paragraphs to be more connected and we hope that the different motivations are conveyed clearly by each paragraph.

3)The methodology must be explained in detail and the author should specify how their approach is different. - We have added more details to the methodology and we hope the details suffice to understand the methods used in quantification of the paper.

4)The results should be compared with existing works. - We would like to thank the reviewer for this comment. The results are quite unique in their own self. However, as suggested by reviewer 1, we have added a comparison table in the discussion section comparing existing works and our work. We have also added a detailed explanation of the same.

5)The threats to validity must also be present in detail - We understand the reviewer’s concerns. We have added the threats to validity in the discussion section in more detail and have also suggested what further studies need to be conducted to invalidate those threats to the work here.

6)The conclusion and the future work should be presented in detail. - Once again, we are grateful to the reviewer for pointing this out. We have edited the conclusion section to bring forth the future prospects of this system as well as plausible extension of the work described.

Deep neural networks to predict diabetic retinopathy , be used to modify the structure of the paper.

Once again, we are very grateful to you for your kind consideration and to the reviewers for diligently pointing out important gaps in our manuscript. We hope you find everything in order.

Yours sincerely,

Siddharth Pattnaik

Corresponding author.
