# Supplementary material for: A novel home-use culture mechanism for identifying microbial load in urine samples
Source: PLoS One. 2023 May 31;18(5):e0285881. doi: 10.1371/journal.pone.0285881 (PMC10231770; doi:10.1371/journal.pone.0285881)
Supplement: Attachment — Submitted filename: Response_to_reviewers_1.docx [file pone.0285881.s004.docx]

Dear Professor Asare,

Thank you so much for giving us an opportunity to make revision to our manuscript. We are really grateful to all the reviewers and editors for their comments and support. We have now addressed the major comments raised by the reviewers and have edited our manuscript accordingly. The changes have been tracked in the revision that we are submitting. In addition, we are also submitting a clean copy of the manuscript with the changes made. We hope that the changes are satisfactory to the editors. Below is a detailed description of each comment and our response to that:

Reviewer #1: The authors have not well addressed all my comments, some important references are missing. The authors should refer to the previous comments for paper improvement again.

We are grateful to the reviewer for their comments. We have now added the references as suggested by the reviewer in their previous comments. We hope that the reviewer finds the references in place.

Reviewer #2: The authors have addressed all the suggestions provided in the previous review. I recommend the article to be accepted

We are really grateful to the reviewer for their previous comments and for recommending our article for publication.

Once again, we are very grateful to you for your kind consideration and to the reviewers for diligently pointing out important gaps in our manuscript. We hope you find everything in order.

Yours sincerely,

Siddharth Pattnaik

Corresponding author.
